# Livestock and environmental characterization of Colombian municipalities: study of vesicular stomatitis

**DOI:** 10.3389/fvets.2024.1323420

**Published:** 2024-03-26

**Authors:** Daniel Magalhães Lima, Diana Carolina Linares Chaparro, Viviana Marcela Mendez Mancera, Jenny Andrea Vela Merchán, Ana Clara Kohara Roman, Lia Puppim Buzanovsky, Ottorino Cosivi, Manuel José Sanchez-Vazquez

**Affiliations:** ^1^Pan American Foot-and-Mouth Disease Center (PANAFTOSA), Pan American Health Organization, Rio de Janeiro, Brazil; ^2^Ministerio de Agricultura y Desarrollo Rural de Colombia – Instituto Colombiano Agropecuario (ICA), Bogotá, Colombia; ^3^School of Veterinary Medicine and Animal Science, University of São Paulo, São Paulo, Brazil

**Keywords:** geospatial analysis, multivariate data analysis, risk-based surveillance, environmental factors, epidemiological surveillance

## Abstract

Amid the surge in data volume generated across various fields of knowledge, there is an increasing necessity for advanced analytical methodologies to effectively process and utilize this information. Particularly in the field of animal health, this approach is pivotal for enhancing disease understanding, surveillance, and management. The main objective of the study was to conduct a comprehensive livestock and environmental characterization of Colombian municipalities and examine their relationship with the distribution of vesicular stomatitis (*VS*). Utilizing satellite imagery to delineate climatic and land use profiles, along with data from the Colombian Agricultural Institute (ICA) concerning animal populations and their movements, the research employed Principal Component Analysis (PCA) to explore the correlation between environmental and livestock-related variables. Additionally, municipalities were grouped through a Hierarchical Clustering process. The assessment of risk associated with *VS* was carried out using a Generalized Linear Model. This process resulted in the formation of four distinct clusters: three primarily characterized by climatic attributes and one predominantly defined by livestock characteristics. Cluster 1, identified as “Andino” due to its climatic and environmental features, exhibited the highest odds ratio for *VS* occurrence. The adopted methodology not only provides a deeper understanding of the local population and its context, but also offers valuable insights for enhancing disease surveillance and control programs.

## 1 Introduction

Different communities, population groups and regions have unique characteristics that directly impact the health and well-being of the individuals within them. Understanding and considering these particularities when planning and implementing health interventions is crucial for the success of health promotion actions. It’s essential to conduct analyses of the local context, considering factors such as demographics, socioeconomic conditions, culture, physical environment, and available healthcare infrastructure. This assessment will identify specific population needs, major health issues, and existing barriers (1, 2). In particular, within livestock populations, characterization has been useful to understand disease-risk differentiation, improve feeding strategies, and define production systems and their valor chain (3–6).

The increasing use of technologies for generating and storing information, including information systems, has led to an exponential rise in the availability of various types of data – environmental, economic, demographic, and epidemiological – in the field of veterinary and public health (7, 8). This growth, stemming from multiple sources and characterized by its vast volume and complexity, presents both new challenges and opportunities for research. In this context, the growing need to integrate advanced analytical techniques to efficiently process and analyze these data becomes increasingly apparent in Official Veterinary Services (OVS) (9). This approach highlights the rising importance of big data in transforming veterinary services, thus opening new pathways to improve health outcomes through well-informed and evidence-based decisions.

Techniques such as large-scale data analysis, machine learning, and data mining have become increasingly relevant. Among these techniques, multivariate analyses such as Principal Component Analysis and Hierarchical Clustering, which use dimension reduction to identify uncorrelated subgroups of variables, have gained prominence (10). These techniques extract valuable insights and support informed decision-making based on available data. Moreover, they play a fundamental role in pattern detection, identification of complex relationships, and generation of knowledge from large volumes of information (10).

*VS* is a disease caused by a vesiculovirus belonging to the Rhabdoviruses family (11). It affects mainly cattle, pigs, and horses and is characterized by ulcerative lesions in the tongue, palate, gums, lips, snout, coronary bands, interdigit, foreskin and teats, a similar clinical presentation to foot-and-mouth disease (FMD). Particularly affecting dairy cattle, *VS* leads to an average morbidity rate of 40% (12) and can cause direct economic losses due to decrease in production, control and prevention measures, estimated at USD 263, per outbreak per farm, in a study by Orrego et al. (13). Moreover, there may be indirect losses due to possible restrictions in animal trade (14) and because it cannot be clinically distinguished from FMD in the field (15).

The epidemiology of *VS* is not yet fully established (11). Some researchers suggest sandflies, black flies and biting midges may be potential vectors (16), or at least a stress factor that favors immunosuppression contributing for clinical appearance of the disease (15). However, there are clear indications that there are at least two distinct major components: the first concerns the disease’s relationship with the environment, particularly the presence of favorable climatic conditions for possible vector proliferation and its association with livestock (17–19); the second component involves animal to animal transmission, occurring both through direct contact and through indirect transmission, via fomites or animal movement (11, 19, 20).

*VS* is widely distributed in the Americas and its prevalence can vary due to factors such as climatic conditions, arthropod presence, and animal management practices (11, 21). In Colombia, it is common for the disease to peak at the end of the rainy season (21), probably due to an increase in vector population and to management practices of herds. Studies between the 1960s and 1980s identified interepidemic periods of 3 years in the country (12), which may occur due to the cumulation of non-exposed animals. One of these studies identified a region of higher incidence and cyclic breakouts - in valleys and specially in foothills and valleys between the Andes - and a region of lower incidence, with geographical and environmental characteristics that differ from the first region (22). Another study observed a significantly higher prevalence in the Aburrá Valley in comparison to highlands and coastal plains (23).

In Colombia, the disease was first reported in Huila, 1929, and Magdalena, 1933. In 1966, *VS* had been reported in most regions of the country and it has been increasing in numbers in recent years (15). Currently, the most affected departments are Antioquia, Boyacá, Santander, Meta, Nariño, Cauca, Cundinamarca, Casanare, Córdoba, Huila, Putumayo, Tolima y Valle (24). Colombia is a country with significant livestock production, ranking third in South America in terms of cattle population, behind Brazil and Argentina (25). At the same time, it is a country with various ecological areas (e.g., coastal areas, Andes mountains, plains, etc.), leading to different productive potential in each region (26). Understanding the nuances of livestock production becomes crucial when delving into the impact of VS.

The main objective of this study is to characterize Colombian municipalities from both ecological and productive perspectives. Additionally, in a secondary step, to study the association of clinical *VS* with this characterization.

## 2 Materials and methods

### 2.1 Variable selection and data sources

For the characterization of municipalities, which are the epidemiological units in this study, risk factors for *VS* associated with both environmental and livestock components were proposed. For environmental characterization, meteorological variables (27) such as precipitation, temperature, solar radiation, etc., and ecological variables (28), as land use and vegetation cover, were considered (Table 1).

**Table 1:** Climatic variables used for the characterization of Colombian municipalities.

Variable	Abbreviation	Unit	Time frame	Source
Altitude	ALTITUDE	meters	2010–2018	WorldClim (27)
Minimum temperature	MINTEMP	°C	2010–2018	WorldClim (27)
Maximum temperature	MAXTEMP	°C	2010–2018	WorldClim (27)
Precipitation	PRECIP	mm/	2010–2018	WorldClim (27)
Solar incidence	SOLARINC	kJ m-2 day-1	2010–2018	WorldClim (27)
Wind speed	WINDSP	m s-1	2010–2018	WorldClim (27)
Highlands agriculture	HIGHAGRI	% of terrain	2009	ESA (28)
Dry tropics agriculture	DRYAGRI	% of terrain	2009	ESA (28)
Umid tropics agriculture	UMIDAGRI	% of terrain	2009	ESA (28)
Water bodies	WATER	% of terrain	2009	ESA (28)
Rainfed croplands	RAINFCROP	% of terrain	2009	ESA (28)
Bare areas	BARE	% of terrain	2009	ESA (28)
Forest regularly flooded	FLOODFOR	% of terrain	2009	ESA (28)
Deciduos forest	DECIDFOR	% of terrain	2009	ESA (28)
Evergreen forest	EVERGFOR	% of terrain	2009	ESA (28)
Herbaceous vegetation	HERBAC	% of terrain	2009	ESA (28)
Shurbland	SHURBL	% of terrain	2009	ESA (28)
Mosaic Cropland/Vegetation	CROPLVEG	% of terrain	2009	ESA (28)
Mosaic Forest/Grassland	GRASSLFOR	% of terrain	2009	ESA (28)
Mosaic Grassland/Forest	FORGRASSL	% of terrain	2009	ESA (28)
Mosaic Vegetation/Cropland	VEGCROP	% of terrain	2009	ESA (28)
Grassland	GRASSLAN	% of terrain	2009	ESA (28)

To obtain information on livestock, data on farm registration by species, the number of rural properties of different types, and the volume of animal movements by municipality were collected, as shown in Table 2. These data were provided by the Colombian Agricultural Institute (ICA), covering the analysis period from 2009 to 2021. This information was extracted from Colombia’s Epidemiological Surveillance and Information System for Vesicular Diseases. This system is part of a national strategy that promotes timely disease detection in primary production, which is essential for the formulation of appropriate prevention, control, or eradication strategies. The system has a technical structure dedicated to the communication of disease suspicions. At the local level, disease notifications are promoted and verified, epidemiological monitoring is conducted, data and samples are collected, information is processed and analyzed, and communication with the regional level is established (25).

**Table 2:** Livestock variables used for the characterization of Colombian municipalities.

Variable	Abbreviation	Time frame	Unit	Source
Bovines	BOVI	2021	# per municipality	ICA
Bovine herds	BOVIH	2021	# per municipality	ICA
Buffaloes	BUFF	2021	# per municipality	ICA
Buffaloes herds	BUFFH	2021	# per municipality	ICA
Goats	GOAT	2021	# per municipality	ICA
Sheeps	SHEEP	2021	# per municipality	ICA
Horses	HORSE	2021	# per municipality	ICA
Swine (familiar production)	SWINEFAM	2021	# per municipality	ICA
Swine herds (familiar production)	SWINEFAMH	2021	# per municipality	ICA
Swine (industrial production)	SWINEIND	2021	# per municipality	ICA
Swine herds (industrial production)	SWINEINDH	2021	# per municipality	ICA
Swine (backyard production)	SWINEBAC	2021	# per municipality	ICA
Swine herds (backyard production)	SWINEBACH	2021	# per municipality	ICA
Outbound animal movements	MOVOUT	2016–2018	# per municipality	ICA
Outbound k-neighbors	NEIGHOUT	2016–2018	# per municipality	ICA
Bovines exported	BOVIOUT	2016–2018	# per municipality	ICA
Buffaloes exported	BUFFOUT	2016–2018	# per municipality	ICA
Swines exported	SWINEOUT	2016–2018	# per municipality	ICA
Sheeps exported	SHEEPOUT	2016–2018	# per municipality	ICA
Goats exported	GOATOUT	2016–2018	# per municipality	ICA
Animal movements to properties	MOVSPROP	2016–2018	# per municipality	ICA
Animal movements to fairs	MOVSFAIR	2016–2018	# per municipality	ICA
Animal movements to slaughterhouses	MOVSSLAUG	2016–2018	# per municipality	ICA
Animal movements to exportation	MOVSEXP	2016–2018	# per municipality	ICA
Inbound animal movements	MOVSIN	2016–2018	# per municipality	ICA
Inbound k-neighbors	NEIGHIN	2016–2018	# per municipality	ICA
Bovines imported	BOVISIN	2016–2018	# per municipality	ICA
Buffaloes imported	BUFFIN	2016–2018	# per municipality	ICA
Swines imported	SWINEIN	2016–2018	# per municipality	ICA
Sheeps imported	SHEEPIN	2016–2018	# per municipality	ICA
Goats imported	GOATIN	2016–2018	# per municipality	ICA

The information related to vesicular disease notifications used in the second part of this study on the association between the disease and risk factors, including positive cases of *VS*, was provided by the ICA.

### 2.2 Environmental data conversion

To obtain environmental data per municipality, geospatial processing of rasterized data, such as satellite images and statistical projections, was used. During this process, pixel values from each raster file were aggregated by municipality polygon, using measures like averages, to the meteorological variables provided by WorldClim (27), or percentage of municipality occupation, to summarize environmental variables provided by ESA (28). This approach allowed associating relevant information about the environmental characteristics of each municipality, providing a more specific view of the environmental factors present in each defined area. For this the “exactextractr” R package was used (29).

### 2.3 Statistical analysis

The initial stage of the characterization process involved using Principal Component Analysis (PCA) to group the previously mentioned variables. Based on the resulting PCA dimensions, Colombian municipalities were clustered using Hierarchical Clustering, fowling the methodology describe by Husson et al. (10). The goal was to form groups (clusters) composed of similar municipalities according to the factors studied while differentiating them from municipalities in other groups. Several numbers of group divisions were attempted to find the number that best described the variability while maintaining a parsimonious number of groups, seeking in that way a balance between the complexity of the results (regarding the number of groups) and the usefulness and easiness of its interpretation. Descriptive statistics were performed to present the variables that showed statistical significance in the V-test at a significance level (*p*-value <0.05). These analyses were performed using the “FactoMineR” package (30).

In a second step, a simple generalized linear model (GLM) assuming a binomial distribution, was used to assess the occurrence of *VS* against the groups resulting from the Hierarchical Clustering. Thus, the data on *VS* events reported versus the total population of farms at municipality level (i.e., epidemiological unit) was utilized as a response variable, while clustering results was modeled as the explanatory variable. Wald tests were used to examine and present the significance (*p*-value <0.05) of each cluster (versus its baseline) in the final model.

All data manipulation and visualization were performed using the ‘tidyverse’ package (31), and spatial data were managed using the ‘sf’ package (32) in the R software environment (33).

## 3 Results

**Figure 1:**
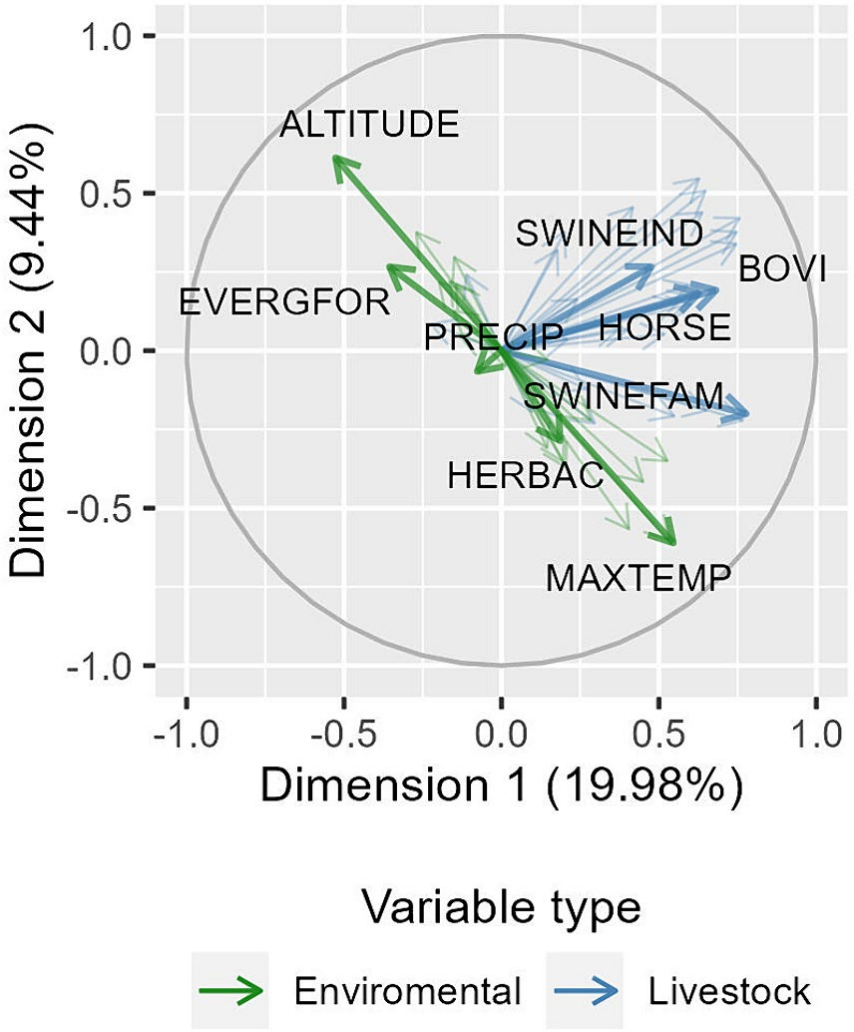
First two dimensions resulting from Principal Component Analysis (axis X and Y respectively). The image presents distribution of all variables used at PCA, thin arrows, and highlights two main axes, one in green, containing mainly variables related to environmental characteristics, and another in blue, composed mainly of variables describing livestock features.

After testing various cluster number combinations (ranging from 2 to 7), forming 4 clusters provided satisfactory differentiation while maintaining a reasonable number of groups. PCA revealed two main evaluation axes in the first two dimensions: one related to environmental characteristics and the other to livestock features. The behavior of these axes led the composition of the formed clusters. The first 5 components explained 74% of the dataset’s variability. In Figure 1, two principal axes of correlated variables can be observed: one axis describing environmental variables in green and another axis, perpendicular to the first, representing livestock variables in blue. For example, an inversely proportional relationship between altitude (ALTITUDE) and maximum temperatures (MAXTEMP) is evident, as well as a weaker inversely proportional relationship between precipitation (PRECIP) and the total number of pigs in industrial systems (SWINEIND). Perpendicular arrows represent the lack of correlation between variables.

**Figure 2:**
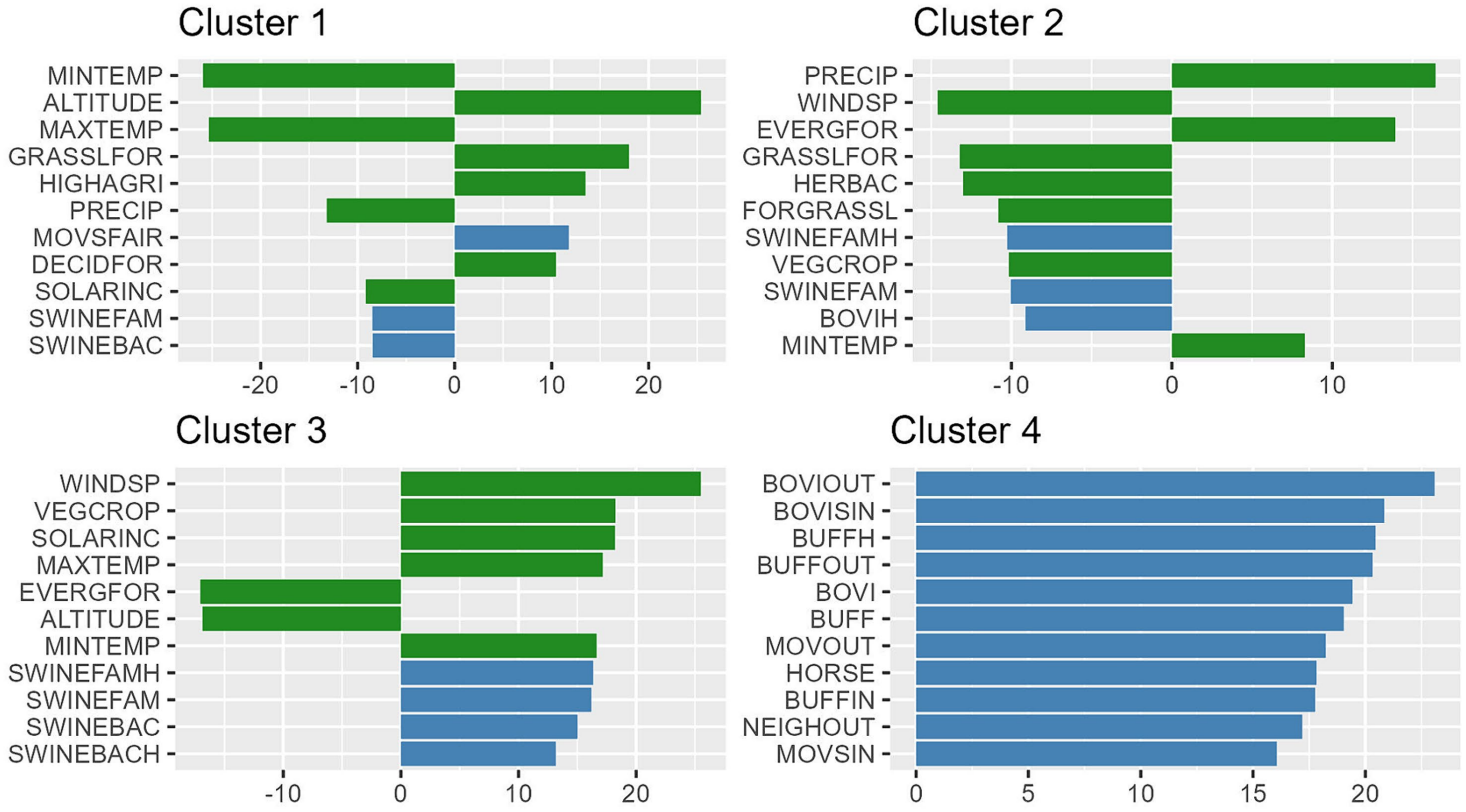
Top 10 significant variables (y axis) in the V-Test (x axis) per cluster (panels). V-Test significance represents the difference between the cluster’s behavior compared to the total municipalities.

A different number of variables contributed to define each cluster; ranging from 36 variables significantly associated at the V-Test for Cluster 4, up to 48 significant variables for Cluster 2. Figure 2 presents the top 10 variables per cluster that contributed most to the distinction between groups. Details of the group characterization can be found in the Supplementary material.

**Figure 3:**
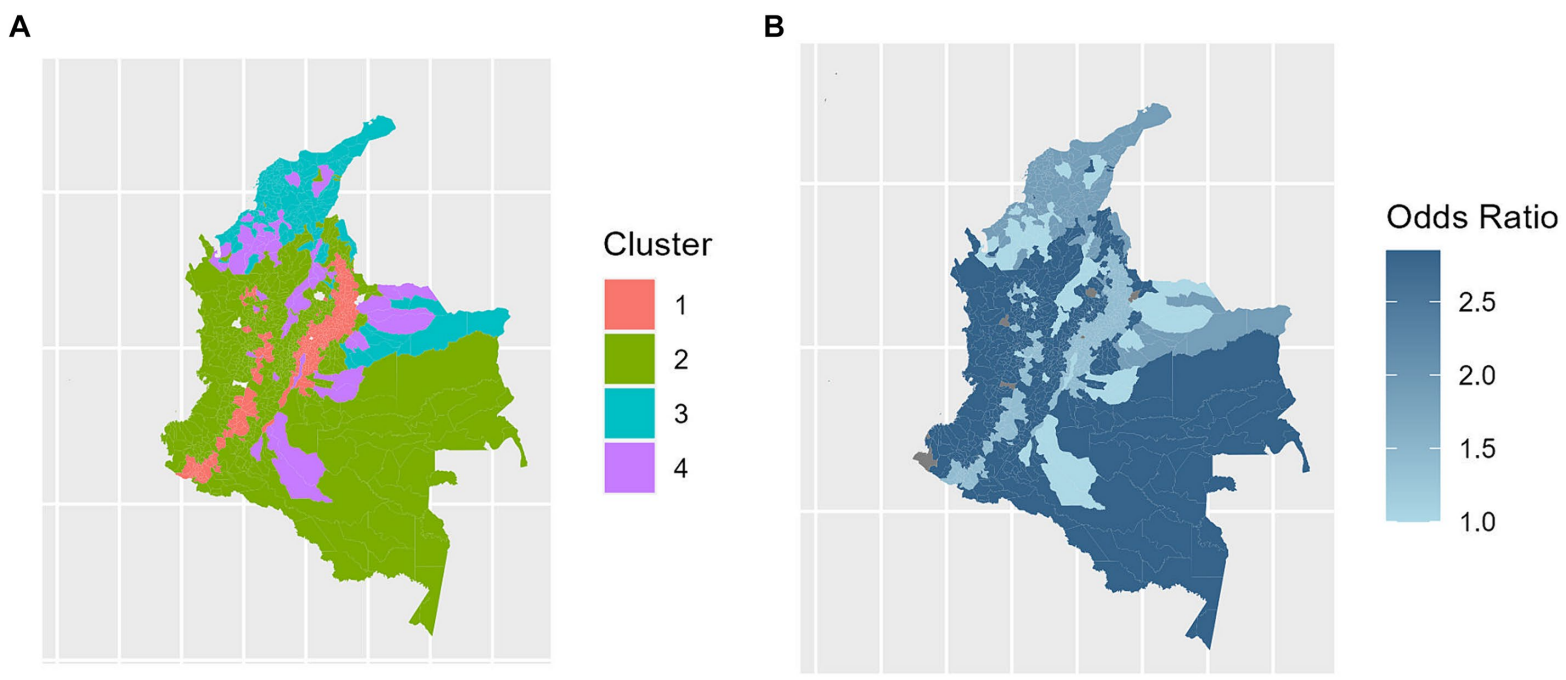
Spatial distribution of the clustering groups **(A)** and relative risk of occurrence of clinical vesicular stomatitis **(B)** in the municipalities of Colombia.

Cluster 1, denoted “Andean,” is characterized by high altitudes, low temperatures, and a significant density of properties, albeit being small-sized ones. Cluster 2, denoted “Amazonian,” stands out for high forest coverage, elevated precipitation levels, and less prominent livestock production. Cluster 3, known as “coastal/Caribbean” covers low-altitude areas with high average temperatures, hosting notable pig production, mainly family-based but with a considerable industrial component as well. Finally, Cluster 4, denoted as “net livestock” included municipalities with intensive livestock production across the entire national territory of Colombia, were identified, irrespective of their environmental characteristics. The spatial distribution of the clusters is shown in Figure 3.

The “Amazonian” cluster, in particular, is characterized by conditions conducive to mosquito proliferation, such as high precipitation, mild temperatures, and increased humidity. These factors contribute significantly to the epidemiology of diseases within this region. It is this cluster that has been identified with a notably higher incidence of affected herds, indicating a substantially elevated risk for the occurrence of disease, as detailed in Table 3.

**Table 3:** Relative risk for vesicular stomatitis and variables used for its calculation, by cluster.

Cluster	Municipalities	Total herds	Affected herds	Odds ratio	*p* value
1	303	231.570	690	1,40 (1,21-1,62)	<0.0001
2	570	289.296	1747	2,85 (2,5-3,27)	<0.0001
3	178	197.863	786	1,87 (1,62-2,16)	<0.0001
4	50	114.150	243	–	–

## 4 Discussion

The main goal of this study was to characterize the livestock productive areas of Colombia through the application of a highly robust data analysis methodology, allowing to enhance the decision-making information process for ICA OVS. Thus, a livestock-environmental characterization was successfully carried out by integrating various available data sources, in line with the continuous technological evolution both globally and in the context of animal health. From this perspective, the methodological approach outlined in this study demonstrated its ability to handle extensive data sets, providing a thorough yet comprehensible characterization of Colombian municipalities from the OVS perspective.

These results allow for a clear, objective distinction between different eco-livestock clusters or zones. From the most livestock-focused zone with a high concentration of cattle and where environmental characteristics do not significantly influence, Cluster 4 – Net Livestock, to zones with low livestock density and extreme altitude and climate, Cluster 1 - Andean. This information is valuable when approaching surveillance or control programs by OVS, allowing for coherent actions based on regional characteristics (9).

This characterization, in fact, could facilitate ICA decision-makers in designing region-specific health strategies tailored to local singularities, including risk-based approached founded on a deep understanding of each region’s intrinsic characteristics. For example, as the Cluster 3 “coastal/Caribbean” stans up due to its relevance on pig production, any health intervention aiming at this population should be carefully targeting the municipalities of this cluster. In fact, a good opportunity is offered regarding the implementation of the ICA’s program to promote good practices on the use of swill feeding in pigs (24).

In the second part of this study, we aimed to test how this prior characterization could contribute to determining *VS* risk in a simple manner. The model successfully differentiated varying risk levels, with Cluster 2 “Amazonian” presenting the highest risk characterized by low altitudes, high precipitation, and low farm density per municipality. Similar ecological patterns, such as Amazonian and Andean areas, have been identified in Ecuador. The authors suggest that different ecosystems may favor distinct pathways of *VS* transmission. In Andean communities, due to the high degree of contact among different farms, transmission is facilitated primarily through animal movement and fomites. In areas such as the Amazon, due to climatic characteristics, transmission through vectors is favored (19, 34). Furthermore, other researchers point to a significant genetic similarity between strains found in Ecuador and those found in Colombia (19). Distinct results were observed in Costa Rica by Rodriguez (18), where moist highlands and dry lowlands areas have been identified as regions with a higher disease prevalence in Costa Rica, both in wildlife and livestock.

The utilization and implementation of satellite sources for environmental and climatic data in this study exemplify the utility of big data to improve risk characterization that enables decision-making in the realm of health, in this case, animal health. This methodology was previously applied to characterize aspects of socio-economic relevance affecting Leishmaniasis occurrence in humans (35). The result of this approach provides a deeper understanding of potential relationships between disease distribution patterns and environmental and geographical characteristics, among others. Additionally, it enables the generation of control, prevention, and monitoring measures through OVS in various geographical zones within a territory that share similar regional features. These features might be related to production systems, health culture, commercialization, and movement, among other factors (36).

Part of the information in this study was source upon the existing data available at ICA. Farm registers might encounter a number of potential drawbacks: imprecision (e.g., the number animals or species present at the farm), incomplete (e.g., some farm are not registered) or outdated (as the population of farms is dynamic). Any flaws regarding these data, however, were assumed to have equal chance to be present across the clusters; expecting, thus, no differential bias across them and minimizing the possibility that these errors might have contributed to a meaningful deviance in the results of clustering. Likewise, passive surveillance data originating from rural producers’ notifications might be imperfect, being potentially affected by a lack of sensitivity due to the underreporting. Besides, the underreporting might be unevenly distributed across the Colombian territory in base of the level of sensibilization of the producers. Therefore, these results should be interpreted in light of the potential limitations associated to the use of passive surveillance and should be regarded as of exploratory nature.

## 5 Conclusion

The technique used in this study proved to be helpful in spatially characterizing multiple factors and exploring relationships between environmental, livestock, and *VS* occurrence variables.

## Data availability statement

The datasets presented in this article are not readily available because, while part of the data used in this research comes from spatial data extraction and analysis methodologies, and this information can be easily shared, part of the information used is property of the Instituto Colombiano Agropecuario and should be requested from them if necessary. Requests to access the datasets should be directed to magalhadan@paho.org.

## Author contributions

DL: Conceptualization, Data curation, Formal analysis, Investigation, Methodology, Software, Visualization, Writing – original draft, Writing – review & editing. DC: Data curation, Investigation, Validation, Writing – review & editing. VM: Data curation, Investigation, Validation, Writing – review & editing. JM: Data curation, Investigation, Validation, Writing – review & editing. AR: Conceptualization, Investigation, Writing – review & editing. LB: Conceptualization, Data curation, Formal analysis, Methodology, Writing – review & editing. OC: Funding acquisition, Project administration, Writing – review & editing. MS-V: Conceptualization, Investigation, Methodology, Supervision, Validation, Visualization, Writing – original draft, Writing – review & editing.

## Supplementary material

SUPPLEMENTARY FIGURE S1 T-values of Significant Variables for Hierarchical Cluster Composition. This graph displays the T-values on the X-axis for variables that showed statistical significance in group composition, arranged along the Y-axis. Panels have been grouped by clusters in columns and variable types in rows for better visualization. A positive T-value indicates a mean greater than the group’s average, while a negative T-value represents the opposite. The absence of a bar indicates equality between the group and the overall mean.

SUPPLEMENTARY FIGURE S2 Number of farms reported positive for vesicular stomatitis by municipality in Colombia. The scale has been converted to a base 10 logarithm for better visualization of the variation between municipalities.
