# Supplementary figures and images for: Livestock and environmental characterization of Colombian municipalities: study of vesicular stomatitis
Source: Front Vet Sci. 2024 Mar 26;11:1323420. doi: 10.3389/fvets.2024.1323420 (PMC11002214; doi:10.3389/fvets.2024.1323420)

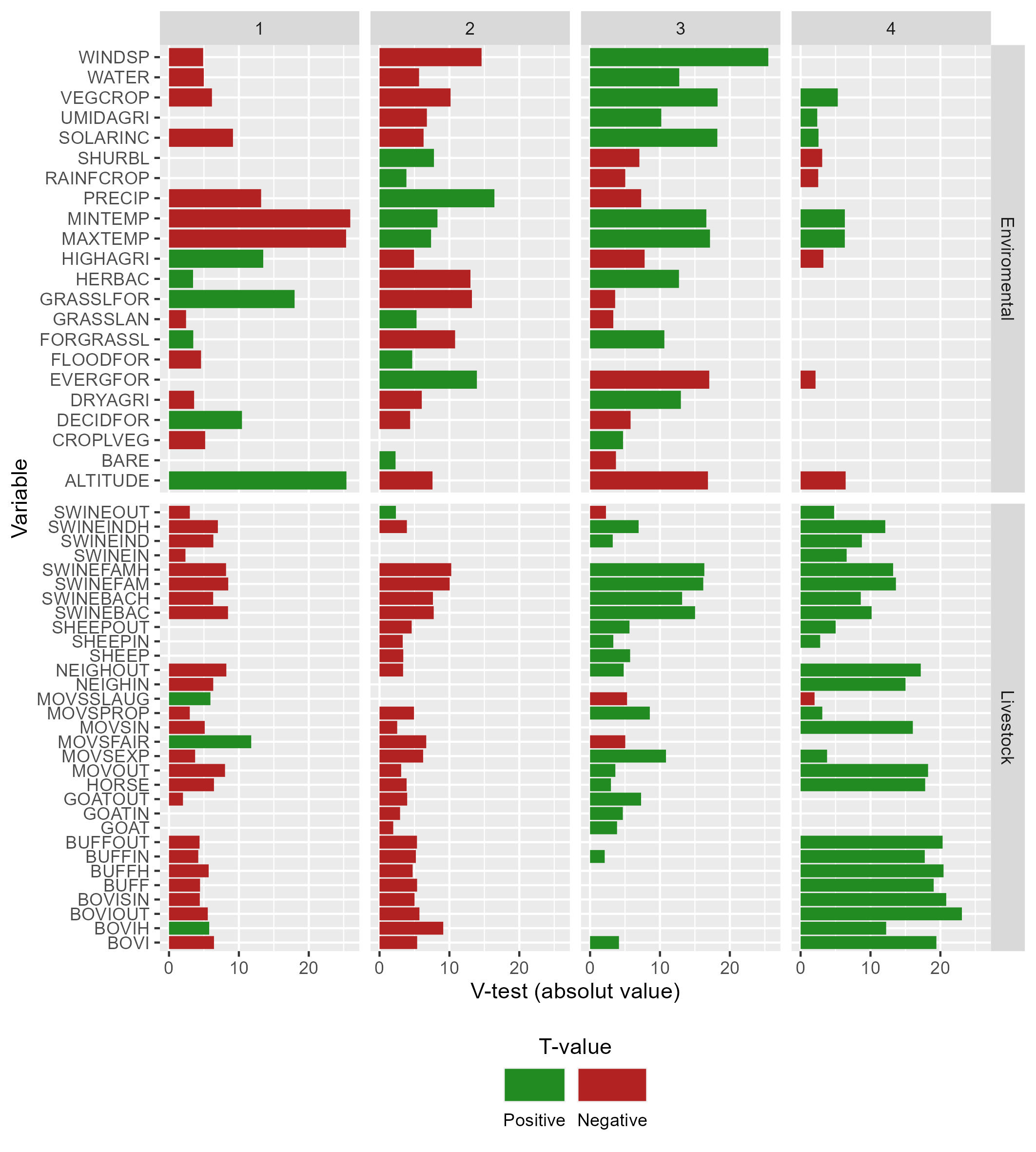

Supplement: SUPPLEMENTARY FIGURE S1 — T-values of Significant Variables for Hierarchical Cluster Composition. This graph displays the T-values on the X-axis for variables that showed statistical significance in group composition, arranged along the Y-axis. Panels have been grouped by clusters in columns and variable types in rows for better visualization. A positive T-value indicates a mean greater than the group’s average, while a negative T-value represents the opposite. The absence of a bar indicates equality between the group and the overall mean. [file Image_1.jpeg]

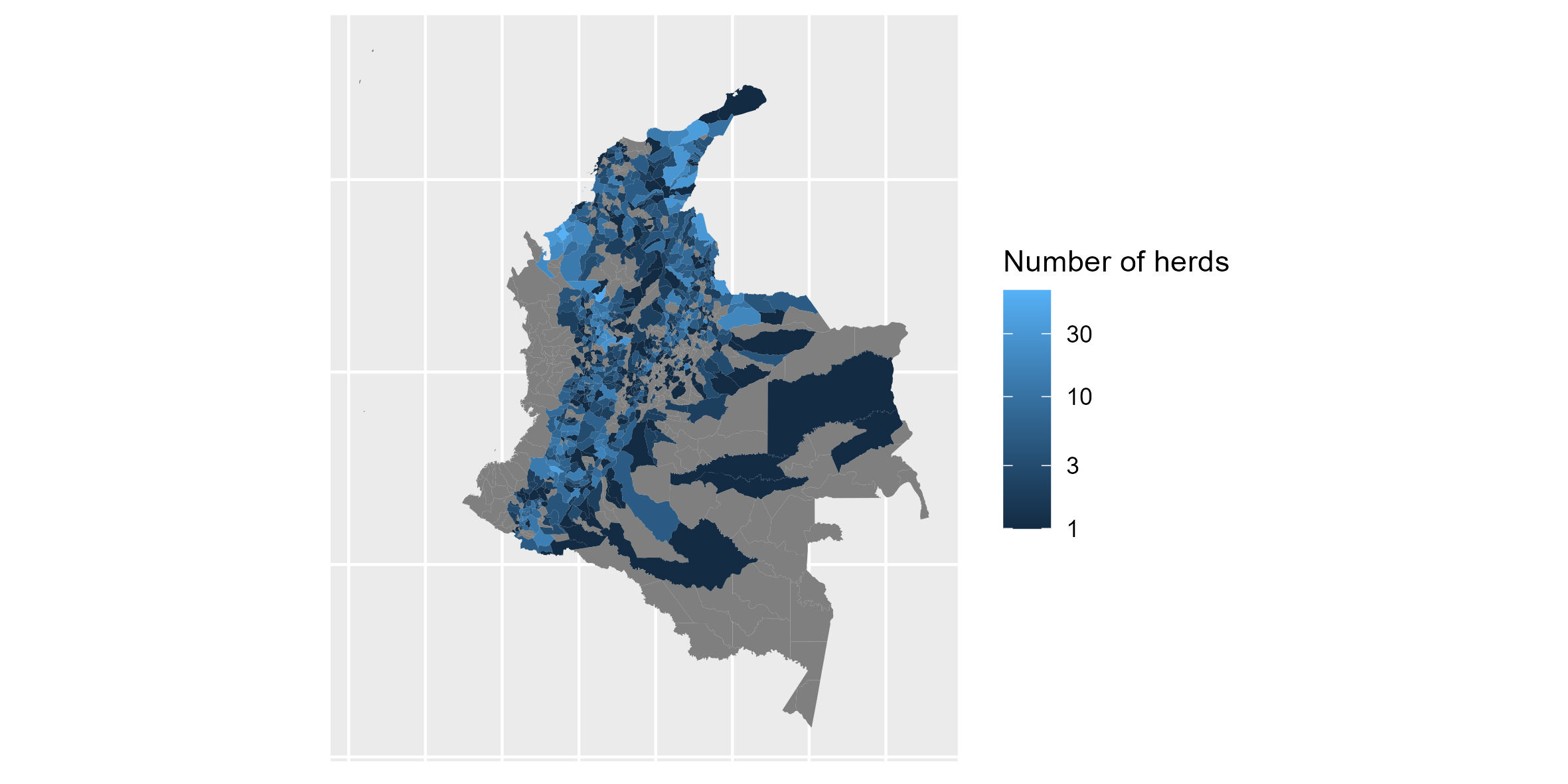

Supplement: SUPPLEMENTARY FIGURE S2 — Number of farms reported positive for vesicular stomatitis by municipality in Colombia. The scale has been converted to a base 10 logarithm for better visualization of the variation between municipalities. [file Image_2.jpeg]
